# Non-competing Trop2 nanobody-based radiotheranostics for precision imaging and therapy of pancreatic cancer

**DOI:** 10.1016/j.mtbio.2026.103230

**Published:** 2026-05-12

**Authors:** Shushan Ge, Jinyu Shi, Tao Xu, Dingding Ai, Meng Zheng, Qingfeng Liu, Yan Wang, Shengming Deng, Liyan Miao

**Affiliations:** a Department of Pharmacy, The First Affiliated Hospital of Sozochow University, Suzhou, China; b Department of Nuclear Medicine, The First Affiliated Hospital of Soochow University, Suzhou, Jiangsu, China; c Institute for Interdisciplinary Drug Research and Translational Sciences, Soochow University, Suzhou, China; d College of Pharmaceutical Sciences, Soochow University, Suzhou, China; e SmartNuclide Biotech, Suzhou, China

**Keywords:** Pancreatic cancer, Trop2, Nanobody, Radiotheranostics, Targeted radionuclide therapy, Trodelvy

## Abstract

Pancreatic cancer remains one of the most lethal malignancies, marked by late-stage detection, resistance to conventional therapies, and dismal overall survival. The transmembrane glycoprotein trophoblast cell-surface antigen 2 (Trop2) is frequently upregulated in pancreatic tumors and drives aggressive phenotypes, positioning it as a compelling target for precision oncology interventions. Here, we reported the development of a novel Trop2-specific nanobody-based theranostic pair, [^68^Ga]Ga-T142 and [^177^Lu]Lu-T142, capable of both non-invasive molecular imaging and targeted radionuclide therapy. The nanobody T142 was conjugated site-specifically with NODAGA or DOTA chelators, preserving its high Trop2-binding affinity alongside exceptional radiochemical purity and stability. In Trop2-positive pancreatic cancer xenograft models, [^68^Ga]Ga-T142 demonstrated rapid and high-contrast tumor accumulation, minimal off-target uptake, and pharmacokinetic profiles characteristic of nanobody tracers. Epitope mapping revealed that T142 recognized a region distinct from that targeted by the clinically approved ADC Trodelvy, allowing accurate, interference-free quantification of Trop2 expression during ADC therapy. Longitudinal PET imaging detected early Trop2 downregulation post-ADC treatment, preceding observable tumor shrinkage, and showed superior performance to [^18^F]FDG in monitoring therapeutic response. On the therapeutic front, [^177^Lu]Lu-T142 elicited marked tumor regression in Trop2-positive models, while its combination with Trodelvy produced synergistic antitumor effects without significant systemic toxicity. Collectively, these results highlighted [^68^Ga]Ga-T142 and [^177^Lu]Lu-T142 as a highly promising Trop2-targeted theranostic platform, with strong potential for precise diagnosis, therapy monitoring, and personalized treatment of Trop2-expressing pancreatic cancer.

## Graphical abstract

**Figure:**
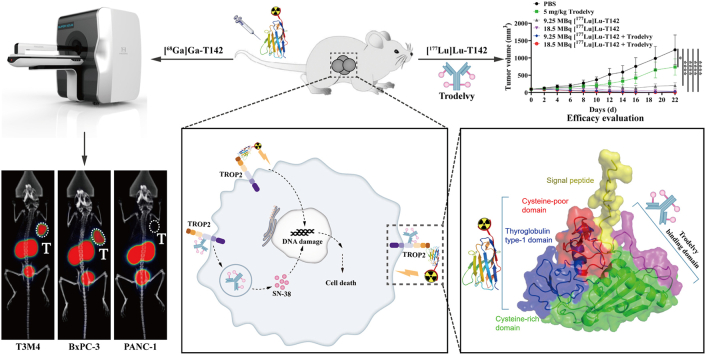

## 1 Introduction

Pancreatic cancer ranks among the deadliest malignancies globally [1]. Recent epidemiological data indicate that it is the sixth leading cause of cancer-related mortality worldwide and the third most common cause of cancer death in the United States [2,3]. Its asymptomatic onset, aggressive nature, and propensity for early metastasis result in the majority of patients being diagnosed at advanced stages [4]. Although multimodal therapeutic approaches have improved disease management, the overall prognosis remains grim, with a 5-year survival rate of approximately 13% [5,6]. This poor outcome is primarily driven by delayed detection, early systemic dissemination, and resistance to conventional treatments [7], underscoring the urgent need for innovative diagnostic and therapeutic strategies to enhance patient survival.

Trophoblast cell-surface antigen 2 (Trop2), a transmembrane glycoprotein encoded by TACSTD2, is overexpressed in numerous epithelial malignancies, including pancreatic cancer, while its expression in normal tissues is largely negligible [8]. Elevated Trop2 levels have been linked to increased tumor proliferation, invasion, metastasis, and poor clinical outcomes, positioning it as a compelling target for precision oncology interventions [9].

Trop2-directed antibody-drug conjugates (ADCs), such as Sacituzumab govitecan (Trodelvy) and datopotamab deruxtecan (Dato-DXd), have shown significant clinical activity in triple-negative breast cancer, metastatic urothelial carcinoma, and non-small cell lung cancer [[10], [11], [12], [13], [14]]. Nevertheless, their efficacy in pancreatic cancer has been limited, largely due to the tumor's complex microenvironment and heterogeneous antigen expression [15]. These limitations highlight the critical need for novel Trop2-targeted strategies that can both accurately quantify Trop2 expression and deliver therapeutic payloads in a spatially precise manner.

In recent years, nanobodies have gained significant attention as versatile agents for immuno-PET imaging of tumor-specific biomarkers, owing to their small size (∼15 kDa), high target affinity, rapid tumor penetration, and swift systemic clearance [16,17]. Their compact structure and adaptable epitope recognition further enable dynamic, real-time monitoring of responses to clinically employed monoclonal antibodies [18]. Beyond imaging, nanobody-based platforms are increasingly being investigated for targeted radionuclide therapy, offering a robust framework for integrated cancer diagnostics and therapeutics in the era of precision oncology.

In this study, we engineered a novel Trop2-specific nanobody, T142, along with its radiolabeled derivatives, [^68^Ga]Ga-T142 and [^177^Lu]Lu-T142, for both non-invasive imaging and targeted radionuclide therapy of pancreatic cancer. [^68^Ga]Ga-T142 enabled high-contrast PET/CT imaging of Trop2-positive tumors, faithfully capturing intratumoral heterogeneity in Trop2 expression. In parallel, [^177^Lu]Lu-T142 demonstrated potent antitumor activity in Trop2-expressing xenograft models, and its combination with Trodelvy produced synergistic therapeutic effects. Collectively, this nanobody-based theranostic approach offered a promising strategy for precise, non-invasive Trop2 quantification and precision-guided radionuclide therapy in pancreatic cancer.

## 2 Materials and methods

### 2.1 Reagents and chemicals

Unless otherwise specified, all reagents and chemicals used in this study were of analytical grade. The bifunctional chelators p-SCN-Bn-NOTA and NODAGA-maleimide were purchased from Macrocyclics. ^68^Ga was obtained from a^68^Ge/^68^Ga generator (Eckert & Ziegler) and eluted with 0.1 M hydrochloric acid. All buffers employed for conjugation and radiolabeling were pre-treated with Chelex 100 resin (Sigma-Aldrich) to eliminate trace metal ions.

### 2.2 Nanobody expression and purification

The Trop2-targeting nanobody T142 was provided by Suzhou SmartNuclide Biopharma Ltd. A glycine-serine-cysteine (GSC) motif was appended to the C-terminus of T142 to facilitate site-specific conjugation with NODAGA-maleimide. The NODAGA-T142 conjugate was synthesized by coupling T142-GSC with NODAGA-maleimide following previously established protocols. The purity and identity of NODAGA-T142 were confirmed using high-performance liquid chromatography (HPLC) and liquid chromatography–mass spectrometry (LC-MS). For radiotherapeutic applications, DOTA-T142 was prepared using an analogous approach, substituting DOTA-maleimide as the chelator precursor.

### 2.3 Surface plasmon resonance (SPR) affinity measurements

The binding affinity of T142 to Trop2 was assessed using a Biacore S200 instrument (Cytiva, USA) equipped with a CM5 sensor chip. Recombinant human Trop2-His protein (Acro Biosystems) was immobilized on the chip at 50 μg/mL in 10 mM sodium acetate buffer (pH 5.0), followed by blocking with 1 M ethanolamine. Serial dilutions of the single-domain antibodies (sdAbs, 0.39–25 nM) were injected with an association phase of 120 s and a dissociation phase of 600 s at a flow rate of 30 μL/min. Regeneration was achieved using 1 mM NaOH. Affinity data were processed and analyzed using Biacore S200 Evaluation Software.

### 2.4 Radiolabeling

To prepare [^68^Ga]Ga-T142, ^68^Ga was eluted from a^68^Ge/^68^Ga generator using 5 mL of 0.1 M HCl. Subsequently, 50 μg of NODAGA-T142 was incubated with ^68^GaCl_3_ in 1 mL solution containing 190 μL of 1 M sodium acetate buffer (pH ≈ 4.5) at 37 °C for 10 min. The radiolabeled product was purified using PD-10 desalting columns with saline as the eluent. For [^177^Lu]Lu-T142, 30 μg of DOTA-T142 was reacted with 740 MBq of ^177^LuCl_3_ in 0.25 M ammonium acetate buffer (pH 7.0) at 45 °C for 30 min. Radiochemical purity and *in vitro* stability were assessed by radio-HPLC on an AdvanceBio RP-mAb Diphenyl column (4.6 × 100 mm), employing water and acetonitrile (both containing 0.1% trifluoroacetic acid) as the mobile phase at a flow rate of 1 mL/min.

### 2.5 Cellular uptake assays

Trop2-positive pancreatic cancer cell lines T3M4 and BxPC-3, along with Trop2-negative PANC-1 cells, were cultured under standard conditions. T3M4 and BxPC-3 cells were maintained in RPMI-1640 medium, while PANC-1 cells were grown in DMEM, all supplemented with 10% fetal bovine serum and appropriate antibiotics. Cell lines were routinely verified to be free of mycoplasma contamination.

For uptake experiments, cells were seeded in 12-well plates and incubated with 37 kBq of ^68^Ga-labeled nanobody for 0.5, 1, and 2 h. In blocking studies, cells were pre-incubated with a 500-fold molar excess of NODAGA-T142. After incubation, cells were washed and trypsinized, and the associated radioactivity was measured using a γ-counter. Uptake was expressed as a percentage of the total added radioactivity (decay-corrected), with each condition performed in triplicate.

### 2.6 Animal models

Female NOD-scidIL2Rgnull (NSG) mice were obtained from Shanghai Model Organisms Center, Inc. Subcutaneous tumor models were established by injecting 1 × 10^7^ T3M4, BxPC-3, or PANC-1 cells suspended in a 1:1 (v/v) mixture of culture medium and Matrigel into female NSG mice. All animal procedures were conducted following the institutional guidelines of Soochow University for the care and use of laboratory animals.

### 2.7 Micro-PET/CT imaging

PET/CT imaging was performed using a Super Nova PET/CT scanner (PINGSENG, China). NSG mice bearing xenograft tumors received 7.4 MBq of ^68^Ga-labeled nanobody via tail vein injection. For blocking experiments, T3M4 xenograft-bearing mice were pretreated with 0.5 mg of T142 prior to tracer administration. Imaging sessions were conducted at 0.5, 1, and 2 h post-injection (p.i.). Quantitative analysis of tracer uptake was performed using region-of-interest (ROI)-based methods for specific organs and tumor tissues.

### 2.8 Micro-SPECT/CT imaging

SPECT/CT scans were acquired using a MARS SPECT/CT scanner (PINGSENG, China). NSG mice implanted with T3M4, BxPC-3, or PANC-1 xenografts were intravenously injected with 11.1 MBq of [^177^Lu]Lu-T142. Imaging was performed at 1, 4, 24, 48, and 72 h p.i. SPECT images were reconstructed and attenuation-corrected using the scanner workstation, and ROIs were delineated for quantitative evaluation of radiotracer distribution.

### 2.9 Biodistribution studies

Biodistribution experiments were conducted in NSG mice bearing subcutaneous T3M4, BxPC-3, or PANC-1 tumors. Mice were injected via tail vein with either ^68^Ga-labeled T142 (∼1.85 MBq in 100 μL saline) or ^177^Lu-labeled T142 (∼7.4 MBq in 100 μL saline). For the [^68^Ga]Ga-T142 cohort, mice were euthanized at 0.5, 1, and 2 h p.i., with blocking studies performed using an excess of unlabeled T142 (0.5 mg per mouse). In the [^177^Lu]Lu-T142 group, animals were sacrificed at 1, 4, 24, 48, and 72 h p.i. Major organs and tumors were harvested, rinsed, weighed, and measured for radioactivity using a γ-counter (2480 WIZARD2, PerkinElmer). Tissue uptake was expressed as the percentage of injected dose per gram (%ID/g).

### 2.10 Epitope mapping and competitive binding with trodelvy

Epitope competition assays were conducted both *in vitro* and *in vivo*. For cell-based experiments, T3M4 cells were pretreated with a 1000-fold molar excess of Trodelvy prior to incubation with [^68^Ga]Ga-T142 (37 kBq). For *in vivo* validation, T3M4 tumor-bearing mice were pre-injected with 1 mg of Sacituzumab or Trodelvy 24 or 48 h before administration of [^68^Ga]Ga-T142, followed by PET/CT imaging and biodistribution analysis to assess competitive binding.

### 2.11 PET-based monitoring of therapeutic response

To evaluate the efficacy of Trop2-targeted ADC therapy, NSG mice bearing T3M4 xenografts were randomly assigned to two groups (n = 14 per group) and treated with either Trodelvy (10 mg/kg, i.p., once weekly) or PBS as a control. Longitudinal PET/CT imaging using [^68^Ga]Ga-T142 and [^18^F]FDG was performed on days 0, 2, 5, 9, and 12 following the initiation of treatment. Tumor uptake of the tracers was quantified as %ID/cc and compared between treated and control cohorts. Selected mice were sacrificed on days 9 and 12 for tumor excision and subsequent histological evaluation using frozen sections.

### 2.12 *Ex vivo* analysis of Trop2 expression during Trop2-ADC therapy

To assess Trop2 expression dynamics in response to Trodelvy, animals were euthanized at predetermined time points, and tumors were collected for histological and immunofluorescence analysis. Tumor samples from both PBS- and Trodelvy-treated groups were formalin-fixed, paraffin-embedded, and sectioned at 5-μm thickness. Sections were deparaffinized and rehydrated through xylene and graded ethanol solutions. For hematoxylin and eosin (H&E) staining, sections were processed using standard protocols and examined under a light microscope. For immunofluorescence, tissue sections were blocked with 5% goat serum albumin (GSA) for 10 min and incubated with a rabbit anti-human Trop2 primary antibody (ab214488, Abcam), followed by a goat anti-rabbit IgG secondary antibody (PV6001, ZSGB-BIO). Cell nuclei were counterstained with DAPI (Beyotime), and fluorescence imaging was performed using a Leica VERSA8 scanner to visualize Trop2 expression.

### 2.13 Radiotherapeutic study

The antitumor efficacy of [^177^Lu]Lu-T142, both as a monotherapy and in combination with Trodelvy, was evaluated in NSG mice bearing T3M4 xenografts. Treatments commenced once tumors reached approximately 100 mm^3^. Mice were randomly assigned to five treatment groups: high-dose [^177^Lu]Lu-T142 (18.5 MBq), low-dose [^177^Lu]Lu-T142 (9.25 MBq), high-dose combination (18.5 MBq [^177^Lu]Lu-T142 + 5 mg/kg Trodelvy), low-dose combination (9.25 MBq [^177^Lu]Lu-T142 + 5 mg/kg Trodelvy), and Trodelvy-only (5 mg/kg). PBS-treated mice served as controls. All therapeutic agents were administered on days 1 and 8. Tumor volumes and body weights were measured and recorded every other day. Tumor volume was calculated using the formula: volume = length × width^2^/2. Humane endpoints were defined as tumor volume exceeding 1500 mm^3^ or the presence of tumor ulceration, at which point animals were euthanized.

On day 22, tumors and major organs (heart, liver, spleen, lung, and kidney) were harvested for histopathological analysis via H&E staining to evaluate treatment-associated toxicity. Additionally, immunofluorescence staining for γ-H2AX was performed on tumor sections to quantify DNA double-strand break (DSB) induction across the various treatment groups.

### 2.14 Statistical analysis

All quantitative data are presented as mean ± standard deviation (SD). Statistical analyses were performed using the GraphPad Prism 8.0 software (GraphPad Software, San Diego, CA, USA, RRID:SCR_002798). A p-value <0.05 was considered indicative of statistical significance.

## 3 Results

### 3.1 Preparation and quality control of [^68^Ga]Ga-T142

**Fig. 1:**
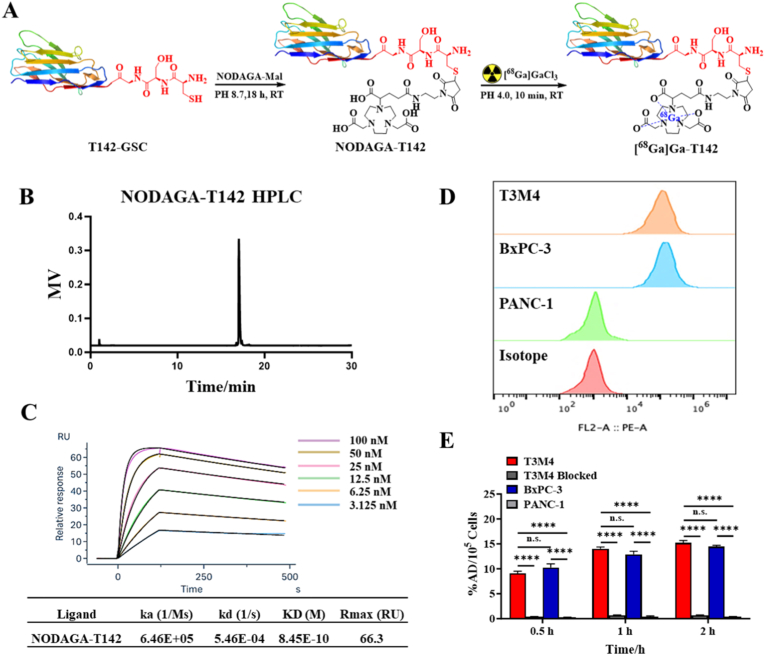
(A) Synthetic scheme for NODAGA-T142 and [^68^Ga]Ga-T142. (B) The purity of NODAGA-142 (C) Binding response (nm) between human Trop2 and different concentrations (3.125 – 100 nM) of NODAGA-T142. (D) The levels of Trop2 expression in various cell lines. (E). Uptake of [^68^Ga]Ga-T142 in T3M4, BxPC-3 and PANC-1 at different times. n.s. indicates no statistically significant difference between groups. ∗∗∗∗P < 0.0001.

The novel Trop2-specific sdAb T142-GSC, engineered with a C-terminal cysteine residue, was site-specifically conjugated with NODAGA-maleimide to produce NODAGA-T142 (Fig. 1A). LC-MS characterization confirmed the precise incorporation of a single chelator molecule per nanobody (Fig. S1A and S1B). HPLC further demonstrated that the purity of NODAGA-T142 exceeded 95% (Fig. 1B).

The binding affinity of NODAGA-T142 toward recombinant human Trop2 was assessed using SPR. The nanobody exhibited a high association rate constant (ka = 6.46E+05 1/Ms) and a low dissociation rate constant (kd = 5.46E-04 1/s), corresponding to a strong equilibrium dissociation constant (KD) of 8.45E-10 M, indicative of robust and stable target engagement (Fig. 1C).

The synthetic procedure for [^68^Ga]Ga-T142 is depicted in Fig. 1A. The radiochemical yield consistently exceeded 95%, with a molar activity greater than 50 GBq/μmol. Stability studies demonstrated that [^68^Ga]Ga-T142 remained highly stable in both saline and human serum, maintaining radiochemical purity above 95% over a 4-h incubation period (Fig. S2).

Flow cytometry analysis confirmed robust cell surface Trop2 expression in T3M4 and BxPC-3 cells, whereas PANC-1 cells displayed negligible expression (Fig. 1D). In cellular uptake assays, [^68^Ga]Ga-T142 exhibited strong, time-dependent accumulation in T3M4 and BxPC-3 cells, reaching 15.20 ± 0.47 %AD/10^5^ cells and 14.45 ± 0.25 %AD/10^5^ cells (n = 4) at 2 h, respectively. Notably, this uptake was significantly blocked by excess unlabeled T142 in T3M4 cells (0.65 ± 0.10 %AD/10^5^ cells), demonstrating high specificity. Similarly, uptake in Trop2-negative PANC-1 cells remained minimal, at 0.35 ± 0.08 %AD/10^5^ cells (n = 4). (Fig. 1E).

### 3.2 ImmunoPET imaging with [^68^Ga]Ga-T142

**Fig. 2:**
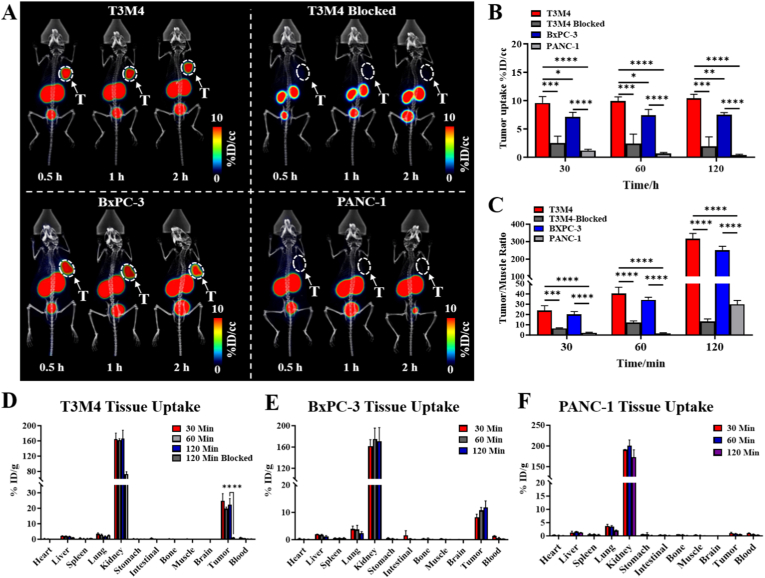
(A) Representative PET/CT images obtained at 30 min, 1 h, and 2 h p.i. of [^68^Ga]Ga-T142 in T3M4, T3M4 Blocked, BxPC-3, and PANC-1 tumor-bearing mice (white arrows indicate tumor lesions). (B) Quantitative tumor uptake of [^68^Ga]Ga-T142 in T3M4, T3M4 Blocked, BxPC-3 and PANC-1 tumor-bearing mice. Values are expressed as %ID/cc. (C) Tumor-to-muscle ratios derived from ROI analysis of PET images. (D) Biodistribution of [^68^Ga]Ga-T142 in T3M4 tumor-bearing mice at 30 min, 1 h, 2 h after injection and at 2 h after co-injected with cold T142 as a blocking agent. Biodistribution of [^68^Ga]Ga-T142 in BxPC-3 (E) and PANC-1 (F) tumor-bearing mice at 30 min, 1 h, and 2 h after injection. ∗P < 0.05, ∗∗P < 0.01, ∗∗∗P < 0.001, ∗∗∗∗P < 0.0001. Data are presented as mean ± SD (n = 3).

Static PET/CT imaging was performed to evaluate the *in vivo* targeting specificity of [^68^Ga]Ga-T142 in NSG mice bearing T3M4, BxPC-3, and PANC-1 xenografts. As illustrated in Fig. 2A, Trop2-positive T3M4 and BxPC-3 tumors were clearly visualized, whereas negligible tracer accumulation was observed in the T3M4 blocking group and in Trop2-negative PANC-1 tumors. ROI-based quantitative analysis revealed substantial uptake in T3M4 and BxPC-3 xenografts (10.36 ± 0.76 %ID/cc and 7.50 ± 0.38 %ID/cc at 2 h, respectively), while tracer uptake in the blocking group and PANC-1 tumors remained minimal (1.93 ± 1.66 %ID/cc and 0.39 ± 0.12 %ID/cc at 2 h) (Fig. 2B). Additionally, [^68^Ga]Ga-T142 exhibited excellent tumor-to-muscle and tumor-to-blood ratios across all imaging time points in Trop2-positive models (Fig. 2C and S3).

These observations were further validated by biodistribution studies, which confirmed the targeting specificity of [^68^Ga]Ga-T142. At 2 h p.i., tumor uptake reached 22.34 ± 3.91 %ID/g in T3M4 and 11.77 ± 2.41 %ID/g in BxPC-3 xenografts, whereas PANC-1 tumors showed minimal accumulation (0.65 ± 0.03 %ID/g) (Fig. 2D–F). Pre-blocking with excess T142 in T3M4 xenografts markedly reduced uptake to 1.11 ± 0.10 %ID/g at 2 h, confirming receptor-mediated binding. Consistent with the renal clearance characteristics of sdAb-based tracers, [^68^Ga]Ga-T142 displayed pronounced kidney retention, with uptake exceeding 150 %ID/g across all models.

To further characterize the *in vivo* pharmacokinetics (PK) of [^68^Ga]Ga-T142, dynamic PET imaging was conducted over 60 min in T3M4 tumor-bearing mice. The tracer demonstrated rapid and pronounced accumulation within tumors within minutes of administration, followed by a gradual increase in concentration (Fig. S4A). Tumor uptake peaked at 18.12 ± 1.70 %ID/cc around 60 min p.i. (Fig. S4B). Among normal tissues, the kidneys and bladder exhibited the highest radiotracer accumulation, whereas activity in the blood and liver steadily declined throughout the scan (Fig. S4C). As systemic clearance progressed, tumor-to-muscle and tumor-to-blood ratios increased over time, reaching maximum values at approximately 120 min, consistent with observations from static PET imaging (Fig. S4D). Consistent with the observed rapid blood clearance PK parameters derived from an image-based blood-pool ROI revealed a circulation half-life (T_1/2_) of 29.03 ± 8.93 min and an area under the curve (AUC) of 327.84 ± 86.74 min·%ID/cc (Fig. S4E).

Immunofluorescence analysis of excised tumor tissues confirmed differential Trop2 expression among the models (Fig. S5). Robust membranous Trop2 staining (green) was observed in T3M4 and BxPC-3 xenografts, whereas PANC-1 tumors displayed minimal signal. The intensity of Trop2 immunostaining closely correlated with tracer uptake in PET imaging, supporting the capability of [^68^Ga]Ga-T142 to non-invasively visualize Trop2 expression in pancreatic cancer. Additionally, H&E staining revealed preserved tumor morphology with no evident necrotic regions.

### 3.3 Epitope mapping of T142 relative to trodelvy

The extracellular domain (ECD) of human Trop2 comprises a cysteine-rich domain (CRD), a thyroglobulin type I domain (TY), and a cysteine-poor domain (CPD). The antibody component of Trodelvy, Sacituzumab, specifically recognizes a linear peptide epitope (Q237–Q252) located within the CPD of Trop2.

To delineate the binding epitope of [^68^Ga]Ga-T142 relative to the therapeutic ADC, a competition assay was performed using Trodelvy (Fig. S6). Pre-incubation with excess Trodelvy exerted minimal impact on tracer accumulation, yielding a 2-h uptake of 16.33 ± 0.60 %AD/10^5^ cells. This value was comparable to the baseline retention (15.20 ± 0.47 %AD/10^5^ cells) established in the cell uptake studies. In sharp contrast, excess unlabeled T142 significantly suppressed uptake to 0.65 ± 0.10 %AD/10^5^ cells. These findings confirm that [^68^Ga]Ga-T142 and Trodelvy engage non-overlapping epitopes on Trop2, highlighting the potential of T142 to quantify Trop2 expression without interference from Trodelvy therapy.

To delineate the binding site of [^68^Ga]Ga-T142, a cell-based blocking assay was conducted using Trodelvy (Fig. S6). The results indicated that pre-incubation with excess Trodelvy had minimal impact on [^68^Ga]Ga-T142 uptake, whereas uptake was markedly inhibited by an excess of unlabeled T142 in an sdAb blocking assay. These findings demonstrated that [^68^Ga]Ga-T142 and Trodelvy engaged non-overlapping epitopes on Trop2, highlighting the potential of T142 to quantify Trop2 expression without interference from Trodelvy therapy.

**Fig. 3:**
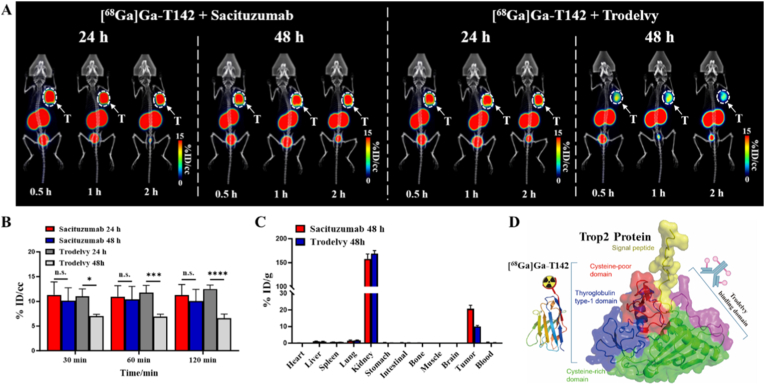
(A) Representative PET/CT images obtained at 0.5 h, 1 h and 2 h after injection of [^68^Ga]Ga-T142 in T3M4 tumor-bearing mice, which had been pretreated with blocking doses of Sacituzumab and Trodelvy either 24 or 48 h prior (white arrow). (B) Tumor uptake of [^68^Ga]Ga-T142 in PET/CT imaging at 0.5 h, 1 h, and 2 h, Values are expressed as %ID/cc. (C) Biodistribution of [^68^Ga]Ga-T142 in T3M4 tumor-bearing mice at 2 h, which had been pretreated with blocking doses of Sacituzumab and Trodelvy 48 h prior. (D) Schematic representation of different Trop2 binding epitopes of T142. n.s. indicates no statistically significant difference between groups. ∗P < 0.05, ∗∗P < 0.01, ∗∗∗P < 0.001, ∗∗∗∗P < 0.0001. Data are presented as mean ± SD (n = 3).

To further validate the distinct epitope recognition of T142 *in vivo*, PET/CT imaging was performed in T3M4 xenograft-bearing mice (Fig. 3A). Mice were pretreated with excess Sacituzumab or Trodelvy 24 or 48 h prior to administration of [^68^Ga]Ga-T142. At 24 h, [^68^Ga]Ga-T142 demonstrated high tumor uptake in both groups (2 h post-tracer injection: Sacituzumab group, 11.22 ± 2.17 %ID/cc; Trodelvy group, 12.48 ± 0.81 %ID/cc) (Fig. 3B). At 48 h, tumor accumulation of [^68^Ga]Ga-T142 remained elevated in the Sacituzumab-pretreated group (10.03 ± 2.39 %ID/cc), whereas uptake was reduced in the Trodelvy-pretreated group (6.60 ± 0.83 %ID/cc).

These PET imaging results were corroborated by biodistribution analysis (Fig. 3C). Two hours after tracer injection, [^68^Ga]Ga-T142 uptake in T3M4 tumors pretreated with Sacituzumab (20.77 ± 2.13 %ID/g) was comparable to that of untreated controls, whereas uptake was significantly diminished in tumors pretreated with Trodelvy (9.90 ± 0.75 %ID/g). Collectively, these findings confirmed that [^68^Ga]Ga-T142 and Sacituzumab recognized distinct, non-overlapping epitopes on Trop2, allowing T142-based imaging to assess Trop2 expression independently of ADC therapy (Fig. 3D).

### 3.4 Monitoring tumor response to trodelvy treatment with [^68^Ga]Ga-T142 PET/CT

**Fig. 4:**
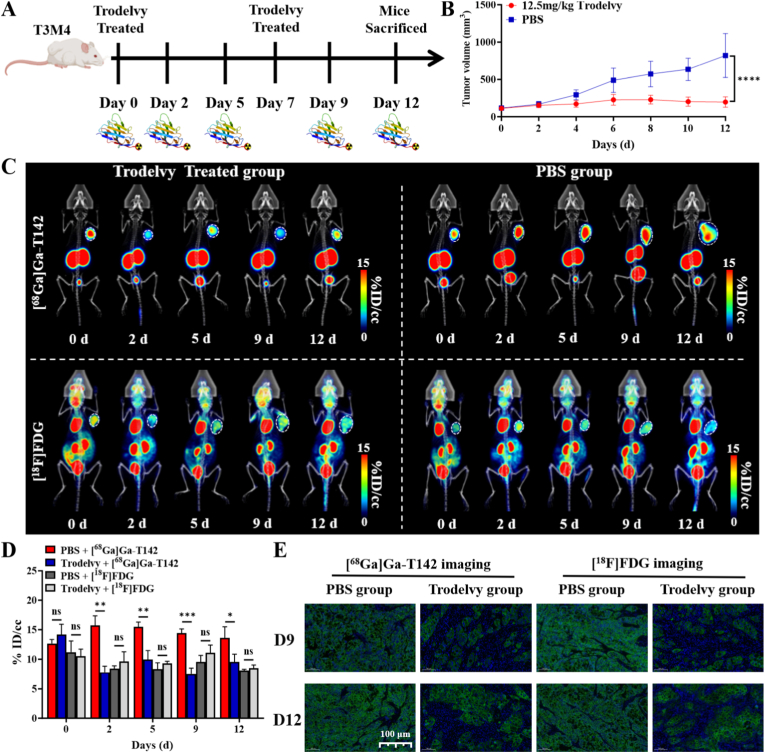
Therapy monitoring of T3M4 tumor-bearing mice treated with PBS and Trodelvy. (A) Experimental protocol. (B) Time-course of tumor volume in T3M4 tumor-bearing mice during treatment. (C) MicroPET imaging of T3M4 tumor-bearing mice at 1 h p.i. of [^68^Ga]Ga-T142 or [^18^F]FDG during PBS or Trodelvy treatment for different durations, respectively. (C) Quantified tumor uptake of [^68^Ga]Ga-T142 and [^18^F]FDG in T3M4 tumor-bearing mice treated with PBS and Trodelvy. (D) Immunofluorescence staining of T3M4 tumor sections during PBS or Trodelvy treatment, respectively (scale bar, 100 μm). n.s. indicates no statistically significant difference between groups. ∗P < 0.05, ∗∗P < 0.01, ∗∗∗P < 0.001.

The therapeutic efficacy of ADCs is closely linked to the expression level of their target antigen, and Trop2-directed ADCs are no exception. To evaluate the treatment response to Trodelvy, [^68^Ga]Ga-T142 PET/CT imaging was employed in NSG mice bearing T3M4 xenografts (Fig. 4A), with [^18^F]FDG PET imaging performed for comparison. Compared with PBS-treated controls, Sacituzumab govitecan treatment produced significant tumor growth inhibition (TGI), achieving a TGI rate of 75.85% (Fig. 4B).

Dynamic microPET/CT imaging was conducted 60 min p.i. of [^68^Ga]Ga-T142 or [^18^F]FDG during the course of therapy. As shown in Fig. 4C, [^68^Ga]Ga-T142 exhibited strong tumor uptake prior to Trodelvy administration, whereas tracer accumulation markedly decreased following treatment, reflecting early modulation of Trop2 expression. In contrast, PBS-treated tumors maintained consistently high [^68^Ga]Ga-T142 uptake throughout the study. Although [^18^F]FDG PET also demonstrated robust tumor accumulation in both treated and control groups, it failed to reveal appreciable changes in tumor uptake over time, underscoring the superior sensitivity of [^68^Ga]Ga-T142 for monitoring Trop2-targeted therapy.

ROI analysis revealed that [^68^Ga]Ga-T142 tumor uptake in the Trodelvy-treated group decreased as early as day 2 after initiation of therapy (day 2: 7.78 ± 1.03 %ID/cc vs. day 0: 14.16 ± 1.78 %ID/cc, P = 0.0006), followed by a modest rebound on day 5 (9.94 ± 1.53 %ID/cc) (Fig. 4D). After the second administration of Trodelvy, a similar trend was observed, with tumor uptake declining on day 9 (7.53 ± 0.95 %ID/cc) and exhibiting a slight increase by day 12 (9.51 ± 1.34 %ID/cc). In contrast, [^18^F]FDG uptake remained relatively stable throughout the treatment course (day 0: 10.51 ± 1.20 %ID/cc; day 2: 9.61 ± 1.64 %ID/cc; day 5: 9.26 ± 0.38 %ID/cc; day 9: 11.07 ± 1.32 %ID/cc; day 12: 8.49 ± 0.53 %ID/cc). In PBS-treated controls, [^68^Ga]Ga-T142 uptake remained consistently high and was significantly greater than in the Trodelvy group at multiple time points (day 2: 15.68 ± 1.68 %ID/cc vs. 7.78 ± 1.03 %ID/cc, P = 0.002; day 5: 15.49 ± 0.80 %ID/cc vs. 9.94 ± 1.53 %ID/cc, P = 0.005; day 9: 14.43 ± 0.70 %ID/cc vs. 7.53 ± 0.95 %ID/cc, P = 0.0005). [^18^F]FDG uptake in PBS-treated tumors exhibited a similar pattern to the Trodelvy group, with no substantial changes over time.

Consistent with PET imaging data, immunofluorescence analysis of excised T3M4 tumors (Fig. 4E) demonstrated strong Trop2 expression in the PBS group throughout the study. In contrast, fluorescence intensity was markedly diminished in the Trodelvy-treated tumors. H&E staining revealed intact tumor architecture in PBS-treated mice, while focal necrotic regions were observed in ADC-treated tumors (Fig. S7). Examination of major organs via H&E staining (Fig. S8) revealed no pathological alterations across all treatment groups, indicating that Trodelvy administration did not induce overt systemic toxicity in NSG mice. Collectively, these findings demonstrated that [^68^Ga]Ga-T142 PET imaging provided a sensitive, non-invasive approach to monitor Trop2-targeted therapy efficacy in pancreatic cancer.

### 3.5 Preparation, SPECT imaging, and biodistribution of [^177^Lu]Lu-T142

**Fig. 5:**
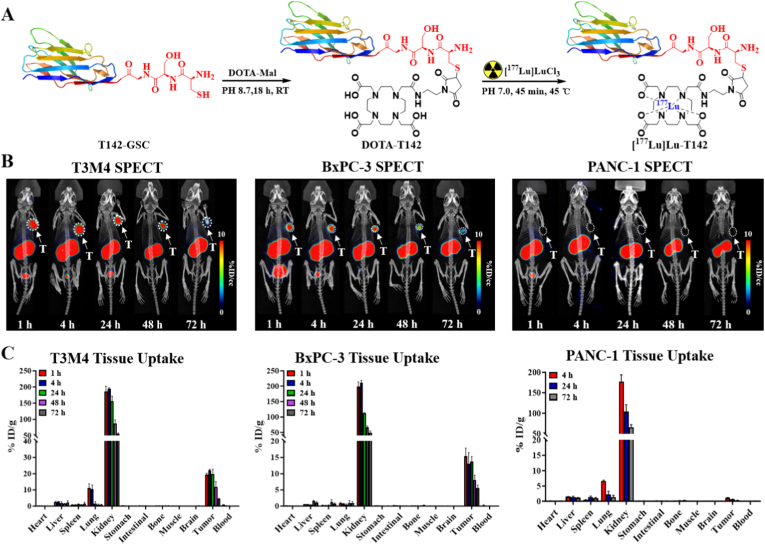
(A) Synthetic scheme for DOTA-T142 and [^177^Lu]Lu-T142. (B) Representative SPECT/CT images obtained at 1 h, 4 h, 24 h, 48 h, and 72 h after injection of [^177^Lu]Lu-T142 in T3M4 tumor-bearing mice (white arrow). (C) Biodistribution of [^177^Lu]Lu-T142 in T3M4, BxPC-3 and PANC-1 tumor-bearing mice at 1 h, 4 h, 24 h, 48 h and 72 h after injection.

The radiolabeling precursor DOTA-T142 was synthesized through site-specific conjugation of the T142-GSC nanobody with DOTA-maleimide, which was subsequently utilized to prepare the therapeutic radiolabeled nanobody [^177^Lu]Lu-T142 (Fig. 5A). LC-MS analysis confirmed precise, site-specific coupling of a single DOTA chelator to each T142 molecule (Fig. S1C). HPLC characterization further demonstrated that the purity of DOTA-T142 exceeded 95%, ensuring its suitability for the preparation of [^177^Lu]Lu-T142 (Fig. S9). Further stability studies demonstrated that [^177^Lu]Lu-T142 remained highly stable in both physiological saline and human serum, maintaining over 90% radiochemical purity even after 72 h (Fig. S10).

SPECT/CT imaging was performed in T3M4, BxPC-3, and PANC-1 xenograft models at 1, 4, 24, 48, and 72 h after intravenous administration of [^177^Lu]Lu-T142 (Fig. 5B). Rapid and pronounced tumor uptake was observed in Trop2-positive T3M4 and BxPC-3 xenografts as early as 1 h p.i., reaching 11.61 ± 3.02 and 7.50 ± 1.37 %ID/cc, respectively. Tumor-associated radioactivity remained high at 4 and 24 h (T3M4: 9.08 ± 2.13 and 7.75 ± 1.81 %ID/cc; BxPC-3: 7.19 ± 1.13 and 5.08 ± 0.85 %ID/cc), indicating sustained intratumoral retention. In contrast, Trop2-negative PANC-1 tumors exhibited negligible uptake throughout the imaging period (0.32 ± 0.03 %ID/cc at 1 h; 0.07 ± 0.01 %ID/cc at 24 h), confirming receptor-specific accumulation (Fig. S11). Although tumor uptake declined at 48-72 h p.i., sustained intratumoral retention was still observed. Compared with the rapid PK typically associated with conventional nanobody-based tracers, this prolonged tumor residence provides a supportive basis for subsequent therapeutic applications.

*Ex vivo* biodistribution analysis corroborated the imaging findings (Fig. 5C). In T3M4 tumors, uptake reached 19.38 ± 0.88 %ID/g at 1 h, peaked at 22.16 ± 0.52 %ID/g at 4 h, and remained elevated at 19.64 ± 3.10 %ID/g at 24 h. Similarly, BxPC-3 tumors demonstrated sustained accumulation (15.32 ± 2.56, 13.07 ± 3.37, and 13.78 ± 1.49 %ID/g at 1, 4, and 24 h, respectively). In contrast, PANC-1 tumors showed minimal tracer uptake (1.05 ± 0.16 %ID/g at 4 h; 0.54 ± 0.12 %ID/g at 24 h), further supporting Trop2-dependent tumor targeting. As expected for sdAb-based radiotracers, [^177^Lu]Lu-T142 was predominantly cleared via the renal pathway, with kidney-associated radioactivity decreasing progressively over time.

### 3.6 Therapeutic efficacy of [^177^Lu]Lu-T142 in pancreatic cancer models

**Fig. 6:**
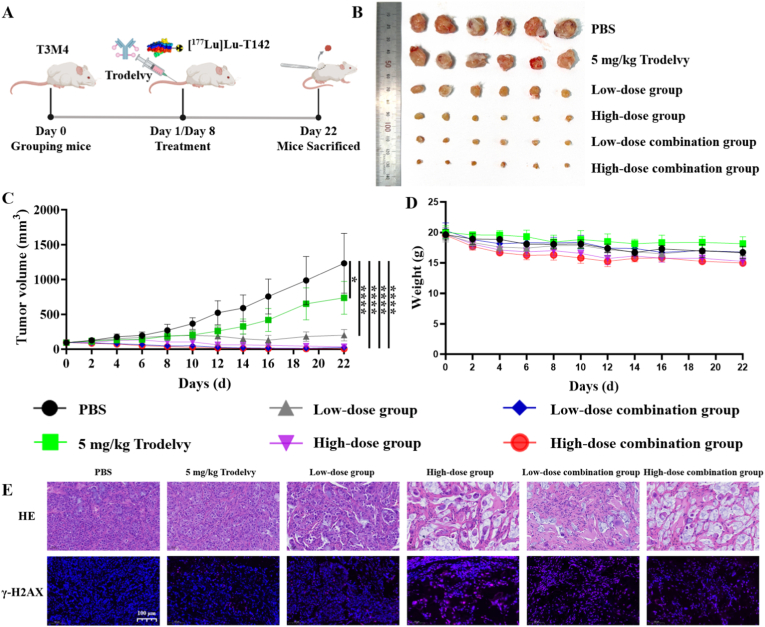
Therapeutic efficacy of [^177^Lu]Lu-T142 alone or combined with Trodelvy in T3M4 tumor-bearing mice. (A) Experimental protocol. (B) Representative *ex vivo* images of tumors collected from each treatment group on Day 22. (C) Time-course of tumor volume in T3M4 tumor-bearing mice during treatment. (D) Time-course of body weight for T3M4 tumor-bearing mice during treatment. (E) H&E and γ-H2AX immunofluorescence staining of T3M4 tumor sections from each treatment group on day 22. respectively (scale bar, 100 μm). ∗P < 0.05, ∗∗∗P < 0.001.

The therapeutic potential of [^177^Lu]Lu-T142, alone and in combination with Trodelvy, was evaluated in NSG mice bearing T3M4 xenografts (Fig. 6A). Mice were randomly assigned to six groups: (i) PBS control; (ii) Trodelvy monotherapy (5 mg/kg, i.v.); (iii) low-dose [^177^Lu]Lu-T142 (9.25 MBq, i.v.); (iv) high-dose [^177^Lu]Lu-T142 (18.5 MBq, i.v.); (v) low-dose combination ([^177^Lu]Lu-T142 9.25 MBq + Trodelvy 5 mg/kg, i.v.); and (vi) high-dose combination ([^177^Lu]Lu-T142 18.5 MBq + Trodelvy 5 mg/kg, i.v.). All treatments were administered on days 1 and 8.

Tumor growth curves revealed continuous enlargement in the PBS group, with volumes surpassing 1000 mm^3^ by day 22 (Fig. 6B and C). In contrast, all treatment arms demonstrated varying degrees of TGI. Trodelvy alone elicited moderate antitumor activity, whereas [^177^Lu]Lu-T142 monotherapy produced pronounced tumor suppression, with the high-dose regimen outperforming the low-dose group. Notably, the low-dose combination regimen achieved tumor regression comparable to that of high-dose [^177^Lu]Lu-T142 alone. Strikingly, the high-dose combination of [^177^Lu]Lu-T142 with Trodelvy yielded the most potent therapeutic effect, resulting in near-complete tumor eradication by day 22. These data underscored the enhanced efficacy of combination therapy, particularly at lower doses, highlighting a clear synergistic effect over monotherapy with either agent.

Body weight monitoring indicated only modest weight loss across all experimental groups. Mice receiving high-dose [^177^Lu]Lu-T142 or its combination with Trodelvy experienced slightly greater reductions in body weight compared with low-dose treatments, yet no overt signs of systemic toxicity were observed (Fig. 6D).

Histological and immunofluorescence analyses of resected tumors corroborated the therapeutic findings. H&E staining revealed extensive necrotic regions in tumors from the high-dose [^177^Lu]Lu-T142, low-dose combination, and high-dose combination groups (Fig. 6E). Correspondingly, γ-H2AX immunofluorescence demonstrated pronounced DNA DSBs in these same treatment arms, reflecting the potent radiobiological effects of the therapeutic nanobody. Notably, H&E evaluation of major organs across all groups showed no pathological abnormalities, confirming that both [^177^Lu]Lu-T142 monotherapy and combination therapy were well tolerated with minimal off-target toxicity (Fig. S12). Collectively, these results indicated that [^177^Lu]Lu-T142, particularly when combined with Trodelvy, produced synergistic antitumor effects in Trop2-positive pancreatic cancer models.

## 4 Discussion

Pancreatic cancer remains one of the most lethal malignancies, largely owing to the lack of effective strategies for early detection and the limited efficacy of current therapeutic options. Its aggressive biological behavior, frequent diagnosis at advanced stages, and complex tumor microenvironment collectively contribute to poor clinical outcomes and present substantial challenges for the identification of actionable molecular targets [5,7]. Among emerging biomarkers, Trop2 has attracted increasing attention due to its frequent overexpression in pancreatic ductal adenocarcinoma (PDAC) and its strong association with tumor aggressiveness and adverse prognosis [19].

Trodelvy, an FDA-approved Trop2-targeted ADC, has demonstrated substantial efficacy across multiple epithelial malignancies, including triple-negative breast cancer and urothelial carcinoma, and is currently being investigated in additional solid tumors [8]. However, the development of Trop2-directed strategies in pancreatic cancer remains relatively limited, highlighting the need for molecular tools capable of both accurately quantifying Trop2 expression and delivering targeted therapeutic payloads. The advancement of such agents may offer a promising avenue for improving precision management in PDAC [[20], [21], [22]].

In the present study, we designed and systematically evaluated two novel Trop2-targeted radiopharmaceuticals, [^68^Ga]Ga-T142 and [^177^Lu]Lu-T142, derived from a newly generated Trop2-specific nanobody, T142. Building upon our previous work, a site-specific conjugation strategy employing NODAGA or DOTA chelators was implemented to enable efficient radiolabeling while ensuring a well-defined and homogeneous molecular architecture. This approach minimized potential interference with the antigen-binding domain, thereby preserving high radiochemical purity and nanomolar binding affinity. Collectively, these attributes supported T142 as a robust and versatile platform for both diagnostic imaging and targeted radionuclide therapy, with considerable potential for further translational development.

Functionally, [^68^Ga]Ga-T142 exhibited rapid and high-contrast accumulation in Trop2-positive pancreatic cancer xenografts, with minimal uptake observed in Trop2-negative tumors or under competitive blockade conditions, thereby confirming its receptor-mediated specificity. The tracer displayed favorable PK characteristics, including rapid systemic clearance and predominant renal excretion, enabling high tumor-to-background contrast within a short time frame p.i.

Importantly, T142 was shown to recognize a distinct and non-overlapping epitope relative to Trodelvy, as demonstrated by both *in vitro* and *in vivo* blocking studies. This property allowed [^68^Ga]Ga-T142 PET imaging to accurately assess Trop2 expression without interference from ADC target occupancy. Longitudinal imaging further revealed that alterations in tracer uptake preceded measurable changes in tumor volume, highlighting its potential as an early biomarker of therapeutic response. These imaging findings were corroborated by histopathological analyses, which demonstrated decreased Trop2 expression and increased tumor necrosis following treatment.

Compared with previously reported radiolabeled monoclonal antibodies for Trop2 imaging, which typically require 24-48 h p.i. to achieve optimal tumor uptake, [^68^Ga]Ga-T142 demonstrated rapid tumor accumulation and fast blood clearance, likely reflecting the intrinsic PK advantages of nanobody-based tracers [20,[22], [23], [24]].

Recent studies have also described Trop2-targeted peptide-based PET tracers across multiple tumor types, which generally exhibit rapid tumor targeting and favorable tumor-to-background contrast [[25], [26], [27]]. However, these agents typically display only moderate binding affinity (in the tens to hundreds of nanomolar range), relatively lower tumor uptake, and rapid washout within 1 h p.i. In contrast, [^68^Ga]Ga-T142 exhibited substantially higher binding affinity (in the picomolar range), along with increased tumor uptake and improved intratumoral retention.

In comparison with previously reported Trop2-targeted nanobody tracers (e.g., [^68^Ga]Ga-NOTA-RTD98, [^68^Ga]Ga-NOTA-RTD161, [^68^Ga]Ga-NOTA-RTD01, [^68^Ga]Ga-NOTA-T4, and [^68^Ga]Ga-MY6349), [^68^Ga]Ga-T142 demonstrated comparable or superior binding affinity and tumor uptake [[28], [29], [30]]. Notably, to the best of our knowledge, T142 represented the first reported nanobody exhibiting a non-competing binding profile relative to Sacituzumab govitecan, thereby enabling the assessment of Trop2 expression during ADC therapy without interference from target occupancy. This feature might offer a distinct advantage for real-time monitoring of target dynamics throughout ADC treatment.

Beyond its diagnostic utility, [^177^Lu]Lu-T142 exhibited potent therapeutic efficacy in Trop2-positive pancreatic cancer models. The radiolabeled nanobody efficiently delivered β-emitting ^177^Lu to Trop2-expressing tumors, resulting in marked, dose-dependent tumor regression. Moreover, combination therapy with Sacituzumab govitecan further enhanced antitumor efficacy, achieving near-complete tumor control in high-dose groups without evident short-term systemic toxicity under the conditions tested. Mechanistic analyses revealed extensive γ-H2AX staining in treated tumors, indicative of widespread DNA double-strand breaks, supporting a complementary mechanism of action in which radionuclide-induced cytotoxicity augments ADC-mediated effects. Collectively, these findings underscored the potential of this nanobody-based platform to integrate molecular imaging with targeted radionuclide therapy for Trop2-positive pancreatic cancer.

Despite these encouraging findings, several limitations should be acknowledged. First, relatively high renal uptake was observed for T142-based radiopharmaceuticals, which is a well-recognized limitation of nanobody-derived tracers, particularly in the context of therapeutic applications. This phenomenon is mainly attributed to glomerular filtration followed by proximal tubular reabsorption. Several strategies have been proposed to reduce renal retention, including modification of linker or tag sequences, co-administration of amino acids or plasma expanders (e.g., succinylated gelatin), and protein engineering approaches aimed at modulating renal handling [18,31,32]. Implementation of these strategies may further improve the safety profile and translational potential of [^177^Lu]Lu-T142.

Second, clinically evaluated Trop2-targeted nanobody tracers, such as [^68^Ga]Ga-NOTA-T4 and [^99m^Tc]Tc-MY6349, have been reported to exhibit physiological pancreatic uptake in humans, potentially reflecting endogenous Trop2 expression in pancreatic tissue [[33], [34], [35]]. Therefore, the applicability of T142-based radiopharmaceuticals for Trop2-targeted imaging and therapy in pancreatic cancer requires further validation in prospective clinical studies to comprehensively assess their diagnostic performance and therapeutic efficacy.

Third, the present study primarily evaluated short-term therapeutic responses and safety within a limited observation period. Extended follow-up, including survival analyses and delayed toxicity assessments, will be required to more comprehensively characterize the durability of tumor control and the long-term safety profile of [^177^Lu]Lu-T142.

In summary, [^68^Ga]Ga-T142 and [^177^Lu]Lu-T142 represented a novel class of Trop2-targeted theranostic radiopharmaceuticals that integrate high-resolution molecular imaging with targeted radionuclide therapy. These agents demonstrated high tumor specificity, favorable PK properties, and encouraging therapeutic efficacy, particularly in combination with existing Trop2-directed ADCs. Collectively, these findings provided a strong rationale for further preclinical and clinical development of this nanobody-based platform for precision diagnosis, treatment monitoring, and individualized therapy of Trop2-positive malignancies.

## 5 Conclusion

This study successfully established a pair of novel Trop2-targeted nanobody-based theranostic radiopharmaceuticals, [^68^Ga]Ga-T142 and [^177^Lu]Lu-T142, capable of enabling both high-precision molecular imaging and effective targeted radionuclide therapy in Trop2-positive pancreatic cancer. [^68^Ga]Ga-T142 provided a sensitive tool for non-invasive quantification of Trop2 expression and early detection of therapeutic responses to ADC treatment, whereas [^177^Lu]Lu-T142 demonstrated potent antitumor efficacy, which was further enhanced through synergistic combination with Trodelvy. Collectively, these findings underscored the translational potential of T142-based radiopharmaceuticals as a versatile platform for precision-guided diagnosis, real-time therapy monitoring, and individualized treatment of Trop2-expressing malignancies.

## Ethics approval and consent to participate

All animal experiments were conducted in accordance with the guidelines of the Institutional Animal Care and Use Committee of the Soochow University.

## CRediT authorship contribution statement

**Shushan Ge:** Conceptualization, Data curation, Funding acquisition, Methodology, Validation, Writing – original draft, Writing – review & editing. **Jinyu Shi:** Data curation, Methodology, Validation, Writing – original draft. **Tao Xu:** Writing – original draft. **Dingding Ai:** Data curation, Investigation. **Meng Zheng:** Data curation, Investigation. **Qingfeng Liu:** Data curation, Investigation. **Yan Wang:** Conceptualization, Methodology, Validation, Writing – review & editing. **Shengming Deng:** Conceptualization, Methodology, Validation, Writing – review & editing. **Liyan Miao:** Conceptualization, Funding acquisition, Supervision, Validation, Writing – review & editing.

## **Declaration of competing interest**

The authors declare that they have no known competing financial interests or personal relationships that could have appeared to influence the work reported in this paper.

## Data Availability

Data will be made available on request.
