# Supplementary material for: Non-competing Trop2 nanobody-based radiotheranostics for precision imaging and therapy of pancreatic cancer
Source: Mater Today Bio. 2026 May 12;38:103230. doi: 10.1016/j.mtbio.2026.103230 (PMC13196437; doi:10.1016/j.mtbio.2026.103230)
Supplement: Multimedia component 1 [file mmc1.docx]

Supporting Information

**Non-Competing Trop2 Nanobody-Based Radiotheranostics for Precision Imaging and Therapy of Pancreatic Cancer**

Shushan Ge ^1,2†^, Jinyu Shi ^2†^, Tao Xu ^5†^, Dingding Ai ^2^, Meng Zheng ^1^, Qingfeng Liu ^1^, Yan Wang ^1^*, Shengming Deng ^2^*, Liyan Miao ^1,3,4^*

1. Department of Pharmacy, The First Affiliated Hospital of Soochow University, Suzhou, China;
2. Department of Nuclear Medicine, the First Affiliated Hospital of Soochow University, Suzhou, Jiangsu, China;
3. Institute for Interdisciplinary Drug Research and Translational Sciences, Soochow University, Suzhou, China;
4. College of Pharmaceutical Sciences, Soochow University, Suzhou, China;
5. Smart-Nuclide Biotech, Suzhou, China.

^†^These authors contributed equally to this work.

*Correspondence:

Yan Wang

0814wangyan@163.com

Shengming Deng

dshming@163.com

Liyan Miao

miaolysuzhou@163.com

**Supplementary Figures**


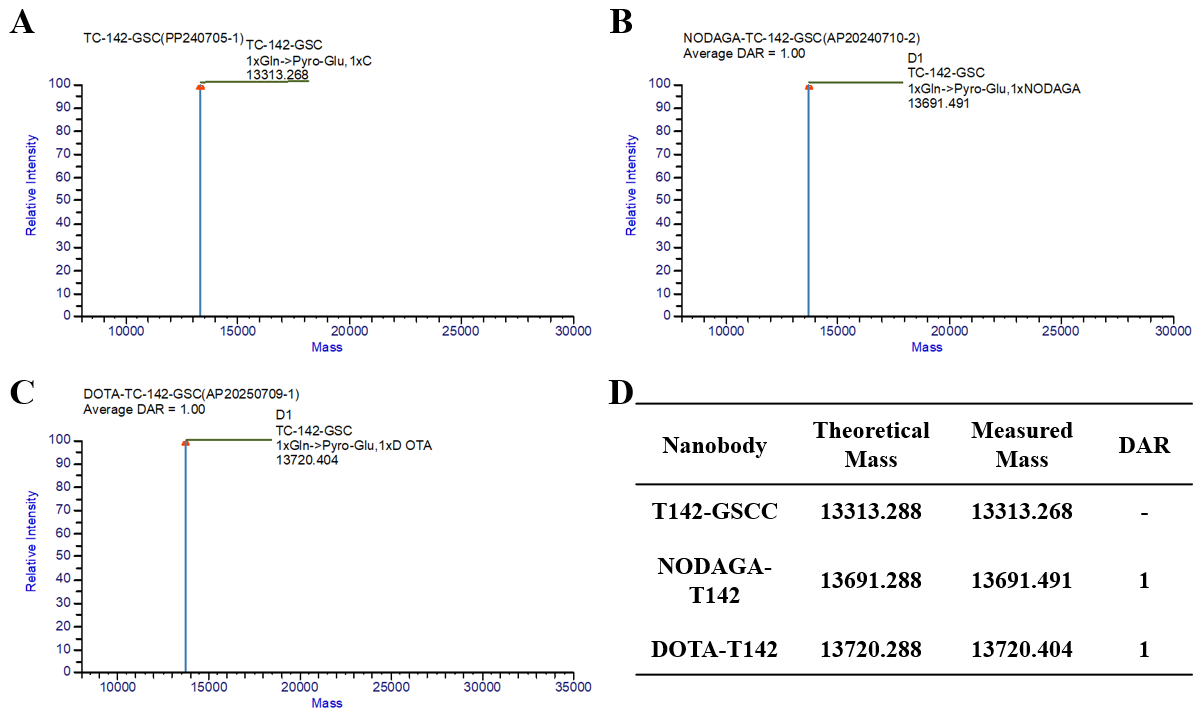


**Figure S1.** Liquid Chromatograph-Mass Spectrometer characterization of T142-GSCC (A), NODAGA-T142 (B), and DOTA-T142 (C). (D) Comparison of the theoretical and experimentally measured molecular weights of T142-GSCC, NODAGA-T142, and DOTA-T142.


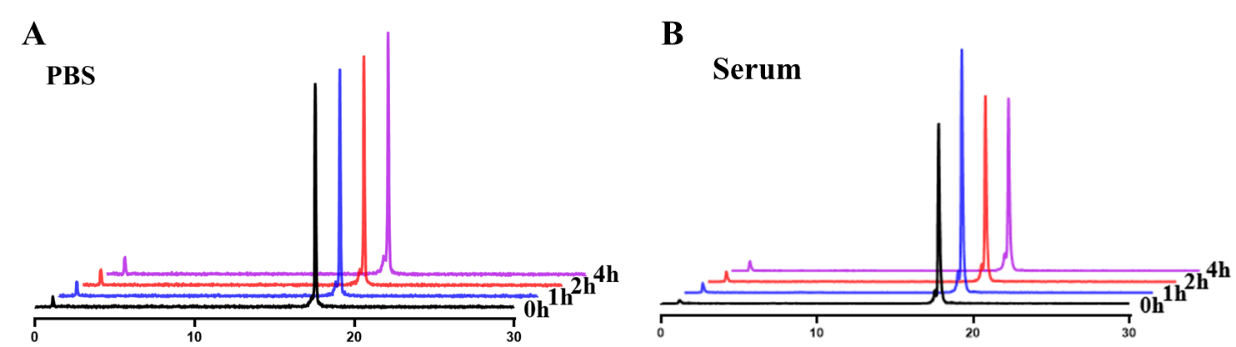


**Figure S2.** The stability of [⁶⁸Ga]Ga-T142 in PBS (A) and Serum (B) at 37 °C measured by radio-HPLC.


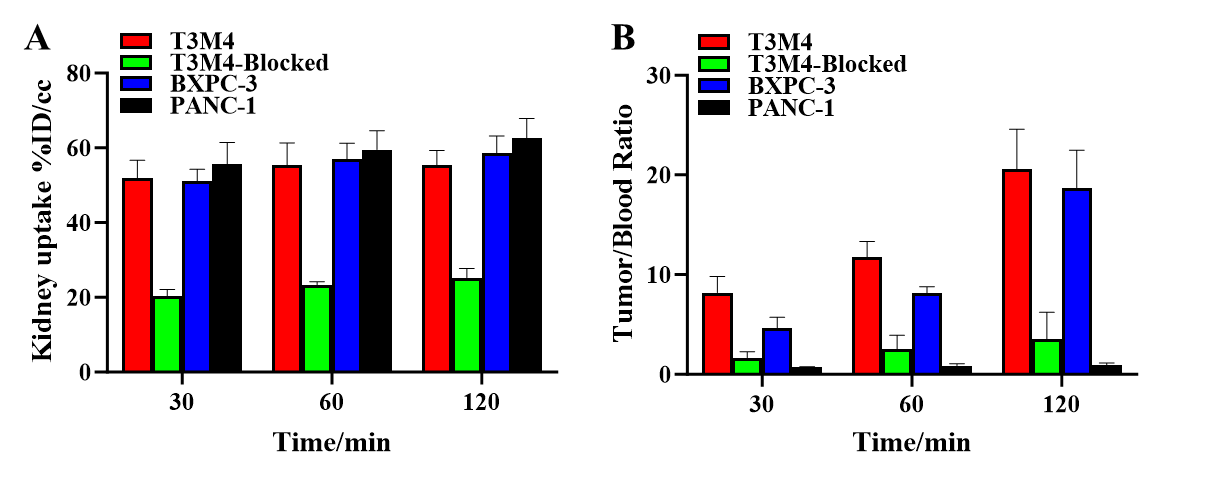


**Figure S3.** (A) Quantitative kidney uptake of [⁶⁸Ga]Ga-T142 in T3M4, T3M4 Blocked, BxPC-3, and PANC-1 tumor-bearing mice. Values are expressed as %ID/cc. (B) Tumor-to-Blood ratios derived from ROI analysis of PET images.


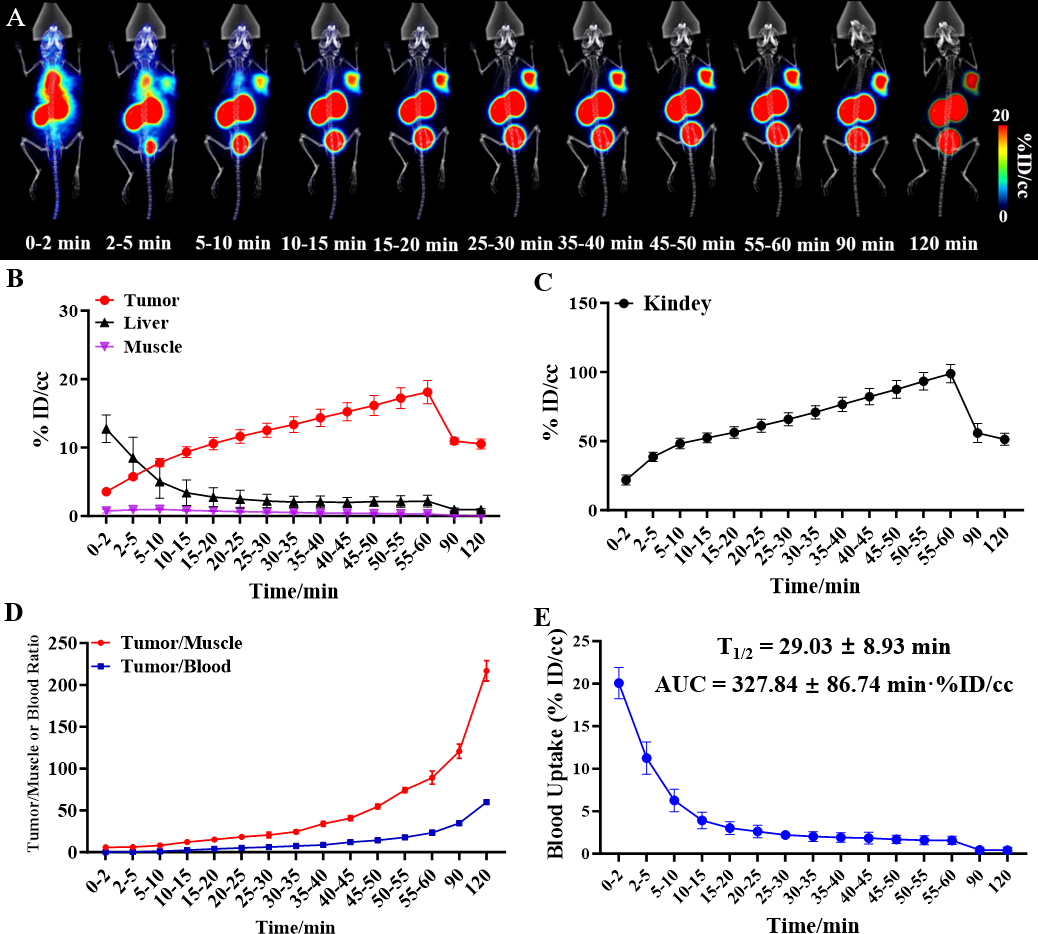


**Figure S4.** (A) Dynamic PET/CT imaging of T3M4 tumor-bearing mice after injection of [^68^Ga]Ga-T142. The tissue uptake (B) and kidney uptake (C) of [^68^Ga]Ga-T142 were analyzed according to the quantification analysis of PET images. (D) The T/M or T/B ratios were calculated according to the quantification analysis of PET images. (E) Pharmacokinetic evaluation of [^68^Ga]Ga-T142 in T3M4 tumor-bearing mice based on dynamic PET/CT imaging.


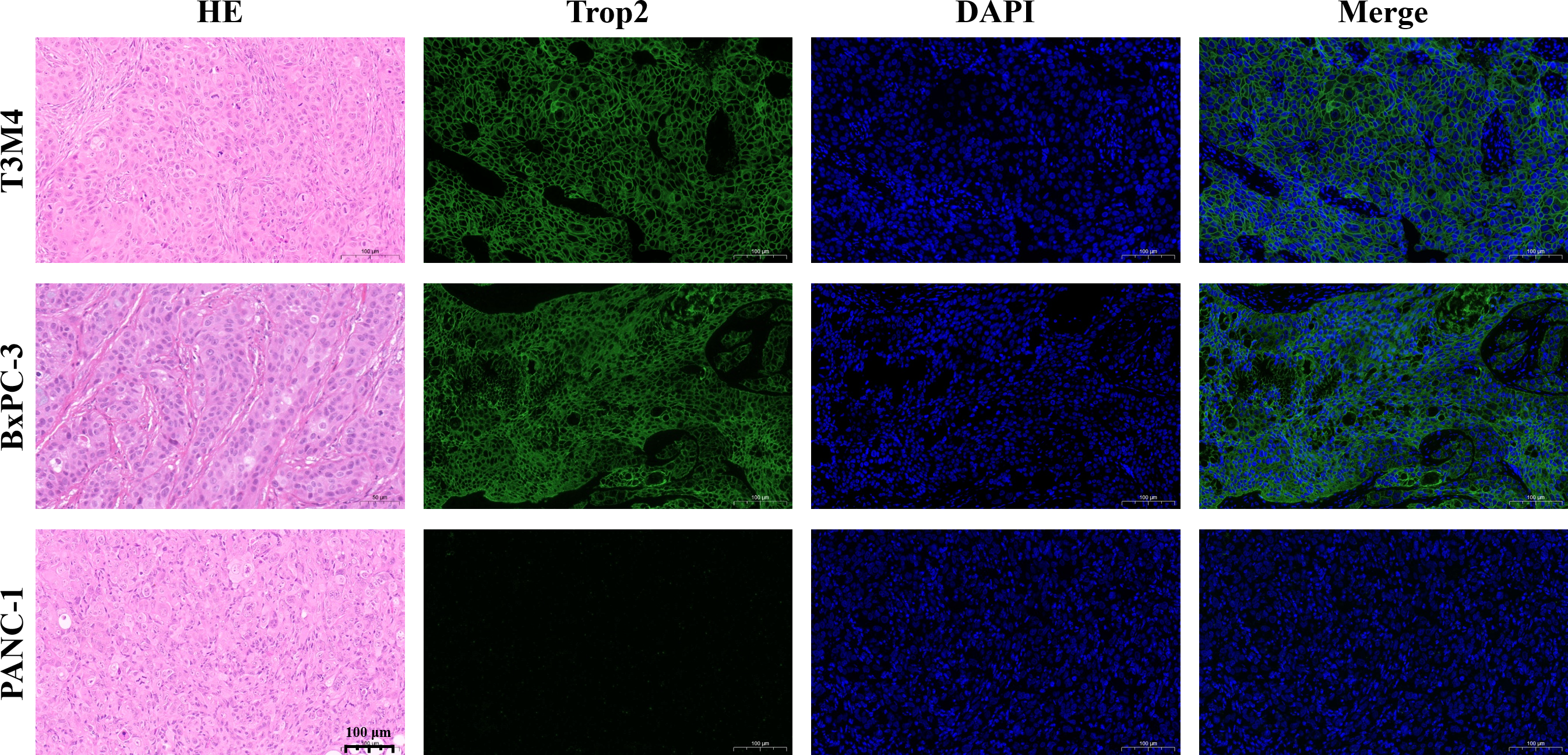


**Figure S5**. H&E and IHF staining of T3M4, BxPC-3, and PANC-1 tumor tissues. Images were acquired at 100 × magnification.

**Figure S6**. Uptake of [⁶⁸Ga]Ga-T142 in T3M4 with or without pretreatment with 1,000-fold unlabeled T142 or Trodelvy at different time points. n.s. indicates no statistically significant difference between groups. ****P < 0.0001.


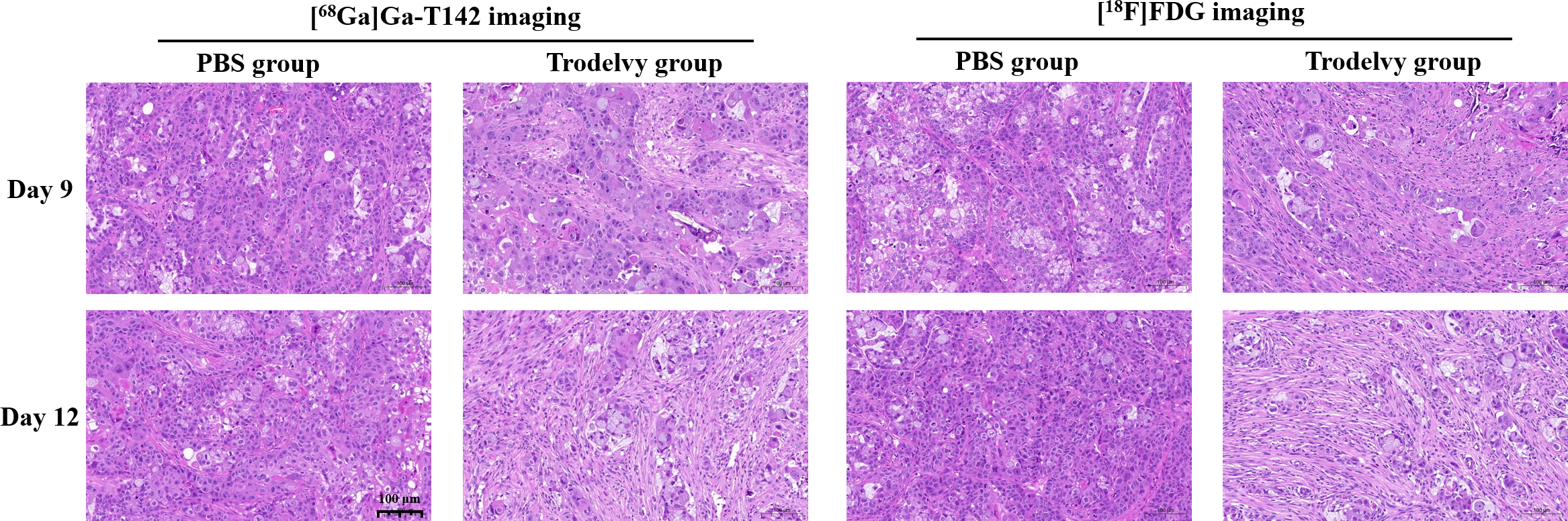


**Figure S7**. H&E staining of T3M4 tumor sections during PBS or Trodelvy treatment, respectively (scale bar, 100 μm).


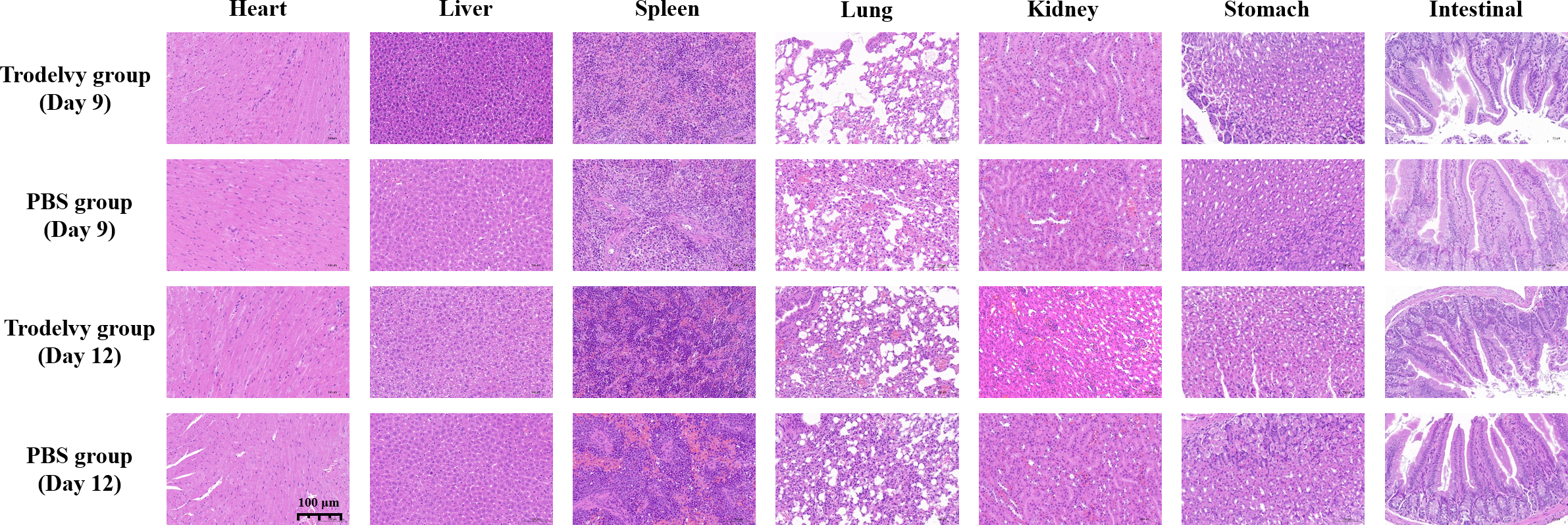


**Figure S8.** H&E staining of normal tissues in T3M4 xenograft tumor models during PBS or Trodelvy treatment, respectively (scale bar, 100 μm).


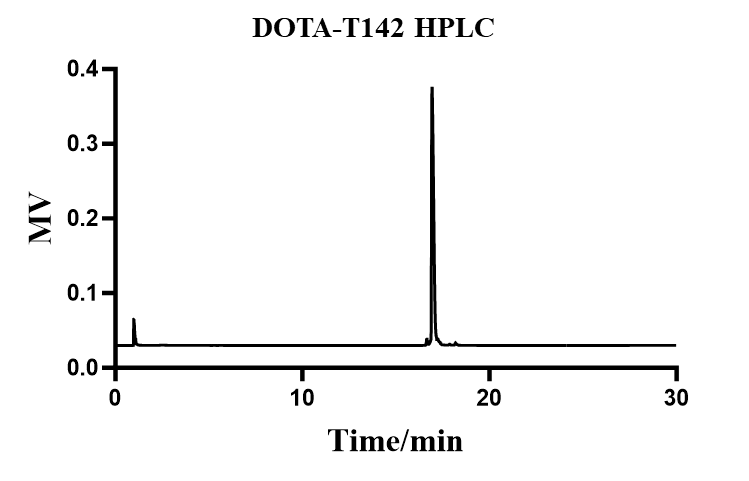


**Figure S9.** The purity of DOTA-T142 determined by HPLC.


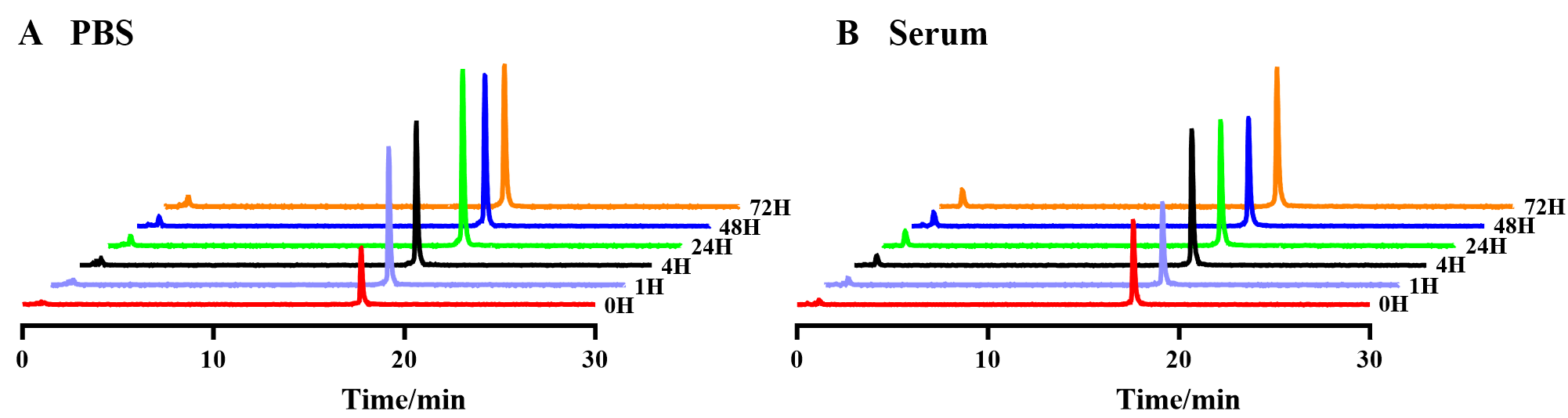


**Figure S10.** The stability of [¹⁷⁷Lu]Lu-T142 in PBS (A) and Serum (B) at 37 °C measured by radio-HPLC.


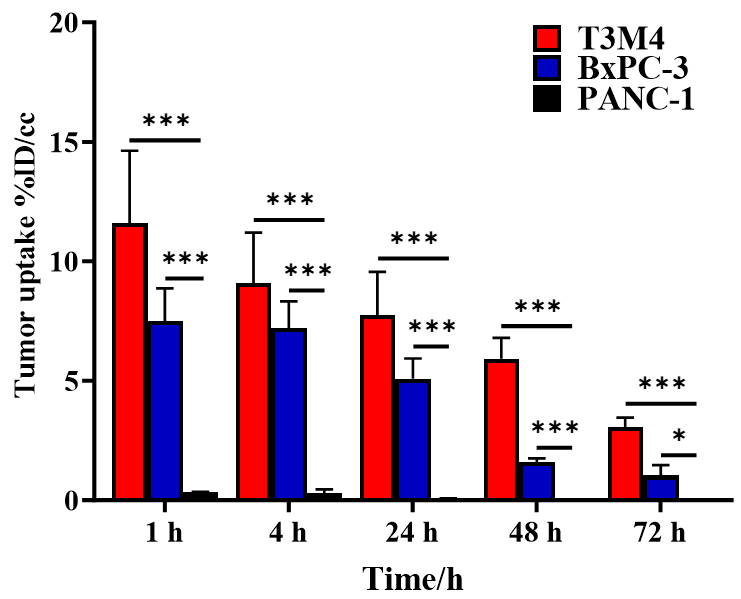


**Figure S11.** Quantitative tumor uptake of [^177^Lu]Lu-T142 in T3M4, BxPC-3, and PANC-1 tumor-bearing mice. Values are expressed as %ID/cc. ***P < 0.001, *P < 0.05.


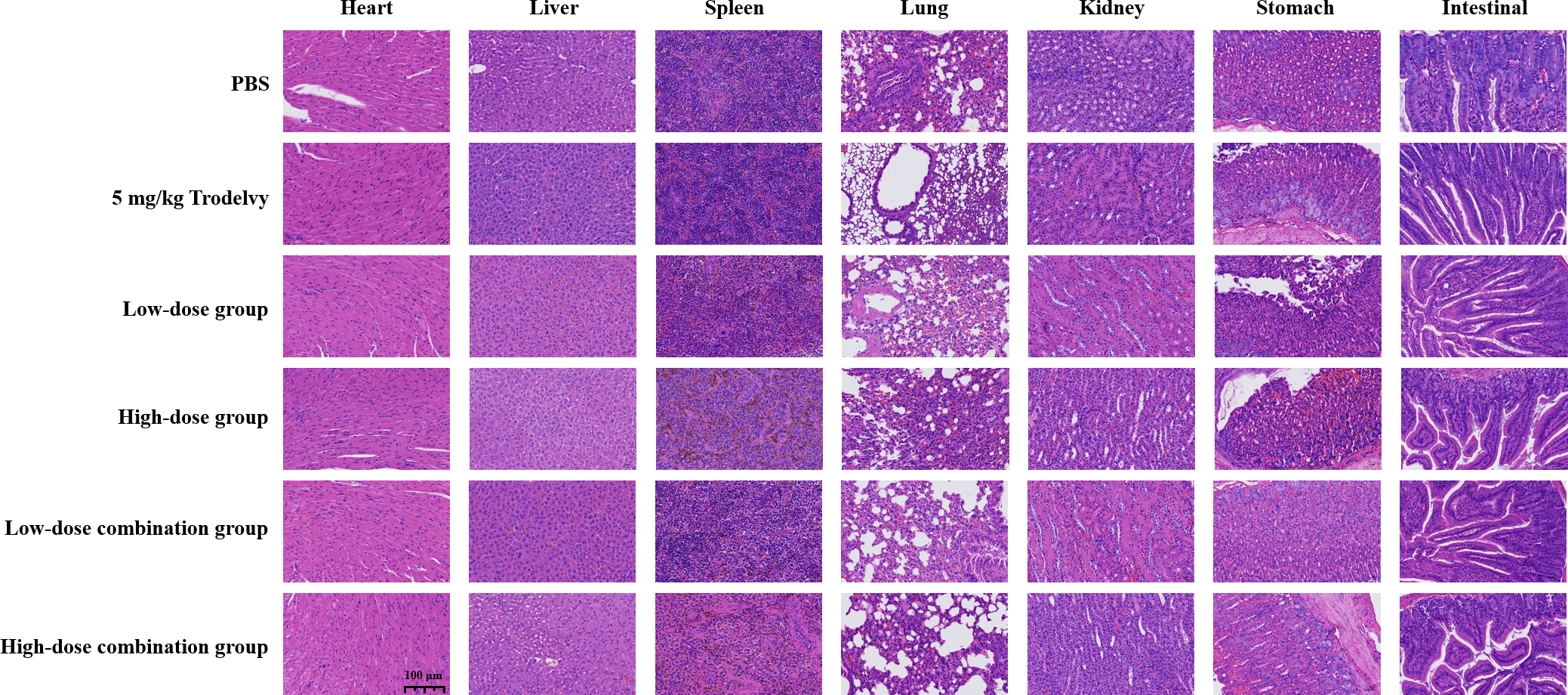


**Figure S12**. H&E staining of normal tissues in T3M4 xenograft tumor models from each treatment group on day 22, respectively (scale bar, 100 μm).
